# Effectiveness of Web-Delivered Acceptance and Commitment Therapy in Relation to Mental Health and Well-Being: A Systematic Review and Meta-Analysis

**DOI:** 10.2196/jmir.6200

**Published:** 2016-08-24

**Authors:** Menna Brown, Alexander Glendenning, Alice E Hoon, Ann John

**Affiliations:** ^1^ Swansea University Medical School Swansea United Kingdom

**Keywords:** acceptance and commitment therapy, systematic review, meta-analysis, depression, anxiety, quality of life, Internet-based, mobile-based

## Abstract

**Background:**

The need for effective interventions to improve mental health and emotional well-being at a population level are gaining prominence both in the United Kingdom and globally. Advances in technology and widespread adoption of Internet capable devices have facilitated rapid development of Web-delivered psychological therapies. Interventions designed to manage a range of affective disorders by applying diverse therapeutic approaches are widely available.

**Objective:**

The main aim of this review was to evaluate the evidence base of acceptance and commitment therapy (ACT) in a Web-based delivery format.

**Method:**

A systematic review of the literature and meta-analysis was conducted. Two electronic databases were searched for Web-delivered interventions utilizing ACT for the management of affective disorders or well-being. Only Randomized Controlled Trials (RCTs) were included.

**Results:**

The search strategy identified 59 articles. Of these, 10 articles met the inclusion criteria specified. The range of conditions and outcome measures that were identified limited the ability to draw firm conclusions about the efficacy of Web-delivered ACT-based intervention for anxiety or well-being.

**Conclusions:**

ACT in a Web-based delivery format was found to be effective in the management of depression. Rates of adherence to study protocols and completion were high overall suggesting that this therapeutic approach is highly acceptable for patients and the general public.

## Introduction

The need for effective interventions to improve mental health and emotional well-being at a population level are gaining prominence globally [1]. These interventions have fast become a priority issue for policy makers in the United Kingdom [2,3] and Europe [4] who have recognized the importance of health and well-being across the lifespan.

Mental health and emotional well-being are a fundamental component of good health. A lack of emotional well-being underpins many physical diseases, unhealthy lifestyles, and social inequalities in health. Common mental disorders (CMDs) such as depression and anxiety are known to be associated with the adoption of unhealthy lifestyle behaviors, including smoking, increased alcohol consumption (above recommended limits), limited physical exercise, and obesity [5,6]. As such, the huge estimated economic costs of these disorders on individuals and society [7] usually associated with lost productivity and burden of health and social services [1,8,9], are likely to be underestimated.

Thus, effective treatments and resources that support individuals to improve their mental health and well-being through psychological treatment programs or health behavior change are of increasing interest to government agencies, health services, commercial enterprises, and individuals themselves. This comes at a time when health and public policy agendas are increasingly encouraging and slowly shifting responsibility for both physical and psychological health, well-being, and lifestyle choice to the individual themselves, so called “self-care” [10,11]. This shift towards personal responsibility is being aided through widespread commercial and technological advancement. Web-based mobile apps that support and encourage healthy lifestyle choices and behavior changes are widely available and affordable [12] and are increasingly utilized for health information [13] and treatment (eg, *Moodgym, fear fighter, beating the blues*). Web-based treatment programs employing cognitive behavior therapy (CBT) are considered to be an effective treatment for a range of conditions, including Post-Traumatic Stress Disorder [14], obsessive compulsive disorder [15], depression [16], anxiety [17], and social phobia [18].

Acceptance and commitment therapy (ACT) has enjoyed a steady rise in interest as an alternative therapeutic intervention to CBT. ACT is considered a third wave CBT, philosophically rooted in functional contextualism [19,20] and relational frame theory [21]. ACT differs from traditional CBT in a number of ways, most notably in that it does not consider thoughts and beliefs as correct or incorrect; and symptom reduction is not the goal of treatment but is a by-product of the process [19]. ACT is based on the principles of self-acceptance and a commitment to one’s personal values; and encourages the adoption of behaviors that are in agreement with those personal values. ACT aims to encourage individuals toward (1) acceptance of difficult and unwelcome thoughts or emotions, and (2) promotion and simultaneous adoption of actions and behaviors, into daily practice, which are in line with these individual core values and principle beliefs. ACT interventions commonly incorporate mindfulness and experiential exercises that promote contact with the present moment.

As interest and research into the application of ACT grows so too must the evaluation of its evidence base. Reviews and meta-analyses have examined the effectiveness of ACT across a range of disorders. Öst [22] concluded that “ACT is not yet well established for any disorder” but showed promise in the treatment of chronic pain and tinnitus with additional possible efficacy for depression, psychotic symptoms, drug abuse, and stress at work. Before this, Ruiz [20] evaluated face-to-face delivery of ACT as compared with traditional (CBT) across a range of conditions and reported a significant mean effect size in support of ACT, for depression and quality of life but not anxiety. Sharp [23] reported that the research base, although small, suggested ACT was effective for a range of anxiety disorders. Powers et al [24] reported a “clear effect and overall advantage of ACT compared to control conditions” but found no evidence to suggest it was more effective than established treatments. Others have also reported ACT to be effective across a range of conditions, including psychiatric disorders [25], chronic pain [26-28], tinnitus [29], multiple sclerosis [30], anxiety disorders [31,32], stress [33-35], and health behavior or lifestyle change together with smoking [36-39] and weight optimization [40]. Thus whilst there is some uncertainty of the effectiveness of ACT, it appears to be related more to establishing the evidence base rather than ACT being an ineffective intervention.

The recent surge in interest in Web-based interventions warrants further review of ACT in the context of a Web-based delivery format to manage CMDs. No previous review has focused exclusively on ACT as implemented in Web format. Although it is important to note that Öst [22] included 3 Web-based ACT interventions. The Association for Contextual Behavioral Science (ACBS) website lists 9 computerized versions of ACT since 2013. Thus, a review focusing solely on this application of ACT is required to assess the evidence base of its effectiveness in the treatment of CMDs.

This review aimed to examine the published, peer-reviewed evidence pertaining to the effectiveness of ACT in the treatment of CMDs and well-being in a Web-based delivery format.

Specific objectives include:

1. Identify randomized controlled trials (RCTs) of Web-based interventions that have employed ACT as the main therapeutic approach, for the treatment of a CMD or improvement of well-being in any population.

2. Appraise and synthesize the evidence on effectiveness for depression, anxiety, and quality of life.

The secondary aim was to report rates of adherence to the study protocol, calculated as a percentage of those randomized to the intervention and completed post assessment.

## Methods

### Search Process

Systemic searches of electronic databases Medline Complete (EBSCO interface) and PsychINFO (EBSCO interface) were conducted from (database inception) to February 10 2016. Standardized subject terms were utilized in each electronic database. The keywords search table can be viewed in Multimedia Appendix 1. The MEDLINE Strategy (EBSCO interface) was adapted for PsychINFO. To address grey literature the list published on the ACBS website of computerized ACT interventions, since 2013, was reviewed. Reference lists of identified studies were examined for additional articles.

Studies were required to meet the following criteria: (1) the study must be published in a peer-reviewed, English language journal (2) the intervention was based on ACT (3) RCT design (random allocation of participants into either intervention and control or intervention and active treatment arm) (4) the study delivers the intervention via the Web (5) the intervention was designed to be accessed on more than one occasion (6) the intervention was designed to manage a CMD or improve well-being and (7) the study must report a measure of effectiveness of the intervention (ie, pre- and post-outcome measure) to enable the calculation of an effect size. Studies were excluded if (1) participants were under the age of 18 years and (2) reanalysis of data from a subsample of a previously published RCT.

### Procedure

Three reviewers (MB, AJ, AG) independently reviewed the title and abstract against inclusion and exclusion criteria. Final decisions were triangulated. Studies were included for full text review where one reviewer indicated to include. The full text article was then assessed against inclusion and exclusion criteria. Studies were excluded when they did not meet a single criterion. The first instance where they did not meet eligibility was recorded and the study was not assessed for other inclusion criteria [41]. In instances where more than one study was retrieved by the same author (MB) checked that the data presented were from different populations. The final list was discussed with the expert reviewer (AJ) to ensure consensus was reached.

### Data Extraction

A data extraction sheet was developed and piloted. The following data were extracted for analysis:

- Citation reference (authors, title and date, country)
- RCT characteristics: RCT design, total number of trial arms, type of comparator or control, method of randomization, allocation sequence concealment, blinding, and sample size and total number allocated to each trial arm
- Participant characteristics: setting (recruited from), condition, comorbidity, diagnostic criteria, age, sex, self-referred or clinician refereed
- Intervention characteristics: name of ACT intervention, specific elements of ACT included, guided or automated delivery, type of guide, type of communication with guide, additional support, intended duration and modules included, format of delivery (sequential or free navigation), features of the system (reminders, personalization)

- Effectiveness of intervention: primary and secondary outcome measures; effect size at post assessment and follow up where available
- Adherence: calculated as a percentage of those randomized to the intervention and completed post assessment.

### Risk of Bias

The Cochrane Collaboration’s tool for assessing risk of bias was applied [41]. A total of 6 risk domains were evaluated. Each domain generated a level of risk: low, high, and unclear, from which an overall level of bias was determined for each RCT. No contact with the publication authors was made to discuss or further clarify points relating to a particular study.

### Analysis

Data were entered into MATLAB, Mathworks and Review Manager (RevMan) version 5.3 (The Nordic Cochrane Centre, The Cochrane Collaboration, Copenhagen), cleaned, and checked for missing values and errors.

### Effect Size Computation

Two categories of effect size were calculated, one comprising between-group effects measured post-treatment and the other comprising within-group effects measured between pre- and post-treatment. In those studies that included more than one comparison condition, the active control was chosen as the comparison condition. For each of these 2 categories, summary effect sizes were then calculated for the following 3 categories of outcome measures: (1) depression (2) anxiety and (3) quality of life. All between-group effect sizes are signed so that a positive value is in favor of the Web-based ACT condition and all within-group effect sizes are signed so that a positive value is in favor of the post-treatment time point of the pre-treatment time point. Hedges’ *g* was chosen for effect size, using means and standard deviations of the outcome measures of participants.

### Meta-Analysis

Analyses were conducted using RevMan version 5.3.5 and MATLAB R2015a. The DerSimonian and Laird random-effects model [42] was adopted in each case, based on the assumption that variation of true effects exists between studies. Using this model, the summary effect sizes outlined in the previous section were calculated. Corresponding tests for statistical significance were computed in the form of both two-tailed P-values and 95% confidence intervals. The heterogeneity of true effects was assessed by the *I*^2^-statistic. Corresponding P-values were computed to assess the extent of uncertainty in *Q*, following the assumption that *Q* follows a *χ*^2^ (k-1) - distribution, with *k*-1 degrees of freedom.

Interpretation of effect sizes was based on Cohen’s rule-of-thumb, that is, small effects were categorized as 0.2 ≤ *g*<0.5, medium effects as 0.5 ≤ *g*<0.8 and large effects as *g*>0.8 [43]. The proportion of dispersion due to true effects was categorized by the well-established scale of Higgins et al [41], that is, the intervals 25% ≤ *I*^2^ < 50%; 50% ≤ *I*^2^ 75% and *I*^2^ > 75% and indicate a low, medium, and high proportion of dispersion due to true effects, respectively.

**Figure 1:**
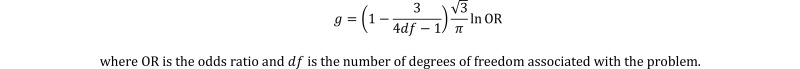
Expression relating the odds ratio and Hedges’ g where df is the number of degrees of freedom.

Where the outcome measure was dichotomized, it was not possible to calculate Hedges’ *g* directly. In this instance, data were transformed using the method given in Figure 1 [44].

## Results

### Principal Findings

**Figure 2:**
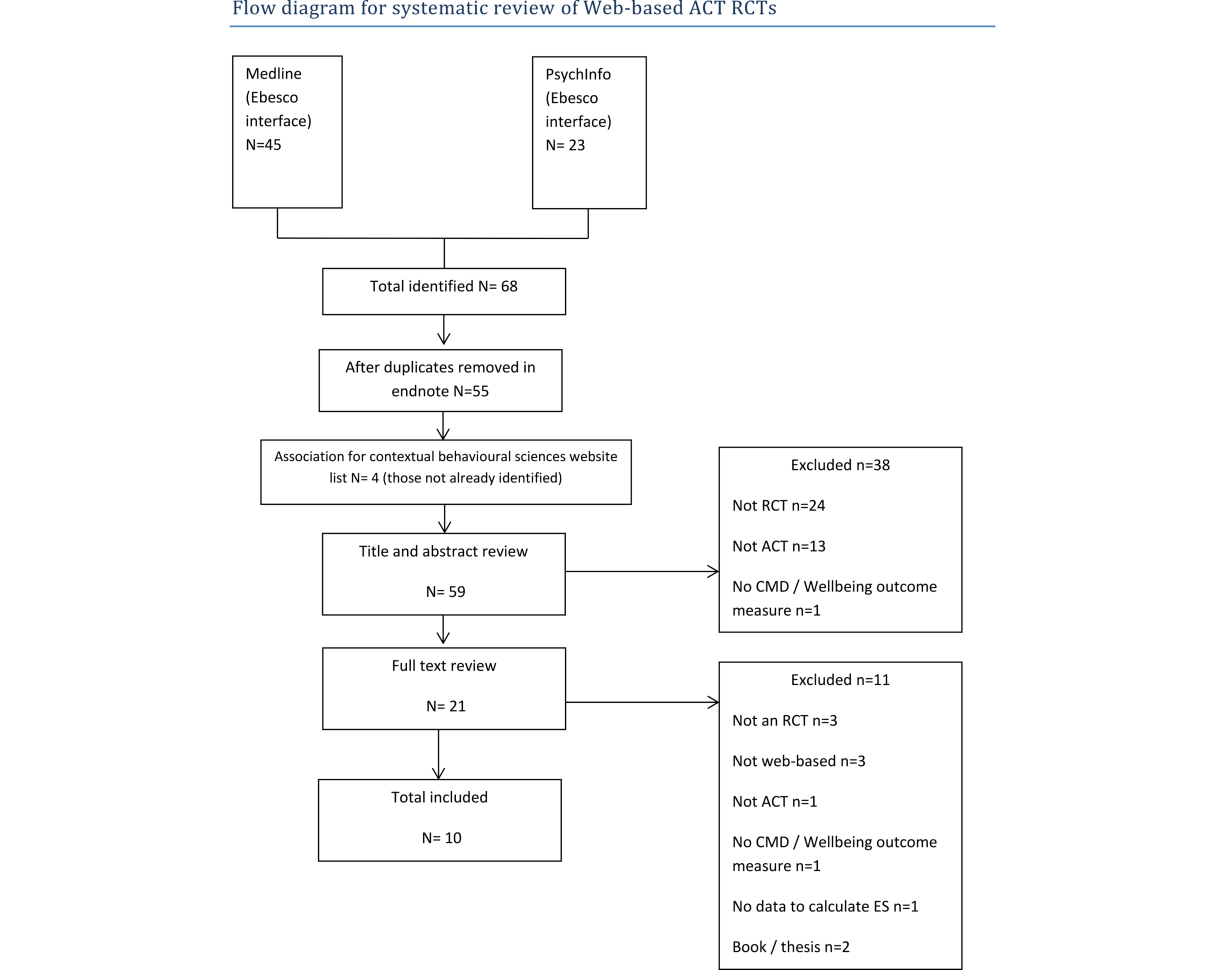
PRISMA flowchart of included studies.

The database searches identified 55 articles; a further 4 were identified through review of the ACBS website. This led to 59 articles being included for title and abstract review. A total of 38 articles were excluded at this stage leaving 21 articles that met inclusion criteria for full text review (Figure 2 PRISMA flowchart). Furthermore, 11 were excluded during full text review, 3 were not RCT design, 3 were not Web-based, 1 did not employ ACT, 1 did not report pre-post outcome for a CMD, 1 did not include data from which an effect size could be calculated, and 2 were not peer-reviewed. Ten RCTs met the full inclusion criteria (Multimedia Appendix 2) and were included.

Two instances were identified where RCTs reported data from the same author [45-48]. In each instance, the 2 reports were assessed. Lappalainen et al [45,46] specified different data collection points, 2011 and 2012 whereas Levin et al [47,48] indicated that participants received different compensation and rewards for taking part (USD$10 plus research credits; USD$60), which suggested different participants were included. For these reasons, each of these articles were included in the review.

Of the 10 RCTs, 7 included 2 trial arms, of which 3 included a wait list control (WLC), and 4 had active controls as the comparator arm. The remaining 3 RCTs included 3 armed trials, of which 2 used an active control plus a WLC and one used 2 active interventions as control (Multimedia Appendix 3). A total of 3 trials were undertaken in Sweden, 3 in the United States, 2 in Finland, and 2 in the Netherlands.

### Participants and Condition

All participants were self-selected. Of the participants, 2 RCTs were recruited from a clinical population (pain clinics) [49,50], 2 from an undergraduate student population [47,48] whereas the remainder were recruited from the general population [29,36,45,46,51]. Trials ranged in size from 38 to 236 participants. Three RCTs included more than 100 participants.

Five interventions were primarily designed to manage and reduce depression and depressive symptoms [36,45,46,51,52] of which one specifically focused on depression in smokers [36], one targeted psychological distress [45], one well-being [46], two chronic pain [49,50], and one tinnitus [29]. All included pre- and post-outcome measures for a CMD, specifically anxiety or depression.

Methods to confirm a diagnosis of primary condition varied. Medical examination and telephone screening [49], computerized screening interview combined with a structured telephone interview [50-52], computerized screening followed by telephone interview and face-to-face meeting plus a medical confirmation of tinnitus [29], self-assessment questionnaires [36], structured clinical telephone interview [45,46], and none [47,48].

### Outcome Measures

A range of outcomes measures were utilized across each RCT which included, Hospital and Depression Scales (HADS), Beck Depression Inventory (BDI), Beck Anxiety Inventory (BAI), Anxiety and depression detector, Depression Anxiety Stress Scales (DASS), and Center for Epidemiologic Studies Depression Scale (CES-D). A range of secondary of measures were also used for quality of life and psychological distress, Quality of Life Inventory (QOLI), general health Questionnaire 12 items (GHQ-12), Symptom checklist 90 items (SCL-90), and the Mental health Continuum Short Form (MHC-SF). A range of ACT specific outcomes were measured in 7 of the included articles, Avoidance and Inflexibility Scale (AIS), Acceptance and Action Scale (AAQ-II), Five Facet Mindfulness Questionnaire (FFMQ), Psychological Inflexibility Scale (PIPS), Engaged Living Scale (ELS), plus an ACT knowledge questionnaire (Multimedia Appendix 4).

### ACT Intervention

Eight separate interventions were identified (Depressionshjälpen, webQuit.org, ACT, Living to the Full, Living with pain, The Good Life compass, ACT-CL, and one unnamed), 2 interventions were utilized in 2 different RCTs (The Good Life compass and ACT-CL). Of which, one was a progression of the first and included additional ACT components [47]. Nine used ACT as the sole therapeutic approach and one [51] used behavioral activation in combination with ACT.

All provided details of the ACT characteristics employed. Four specifically stated that they included modules that attended to all of the 6 core principles of ACT [36,45,50,52]. Two studies used 4 core principles [46,49]. Three studies employed 3 core principles [29,47,51]. One study used 2 core principles [45]. The two Levin et al studies [47,48] ACT-CL were described as prototype interventions exploring feasibility and acceptance. All included mindfulness, experiential exercises, or metaphors, whereas 4 stated they included a maintenance plan for participants (Multimedia Appendix 5).

A total of 3 interventions were automated, that is, no guide or coach was involved in the delivery of the therapeutic program [36,47,48] and the remainder were guided interventions (therapists or coaches assisted and supported participants throughout the delivery of the intervention program). Of the 7 guided interventions, the “guide” was a combination of trained psychologists and psychology graduate students (n=2) or graduate psychology students (n=5). Two of the interventions provided additional support to the clinical guide. One included an administrator and the other a computer technician, both could be contacted if required. The guides provided clinical support in a variety of ways, written secure messages and feedback via the system (n=5), written feedback via email (n=2) and verbal communication over the telephone (n=3). Of which, one was to deliver support and guidance and two acted as reminders to complete the next module in the program. All contact was asynchronous. Of the 3 automated interventions, 2 stated that they provided automated feedback. One intervention provided an additional workbook and a CD, one included a face-to-face meeting at the start and end of the treatment, and one reported a face-to-face meeting prior to commencement of intervention. A total of 6 of the 7 RCTs identified the number of therapists involved in the delivery of the ACT intervention, therapist numbers ranged from 2 to 18.

Intended duration of ACT interventions varied between 3 and 12 weeks in length (M=7.4, SD=3.06) and interventions included between 2 and 9 modules (M=6.4, SD= 2.5).

### System Features

In a Web-based context, the features incorporated into the design of the intervention and computerized system, are of interest as they have the potential to influence engagement and adherence [53]. Email reminders were included in 4 RCTs [46-48,52], short message service reminders [45,48,49,52] and homework tasks were incorporated into 6 RCTs [29,45,47,49-51]. Progression through the program was controlled by either the system or the guide, depending on successful completion of prior modules in 7 systems. Telephone reminders were used in 3 guided interventions to prompt use and encourage continued engagement with the program [46,47,49], personalized feedback on receipt of homework assignments was provided in 5 of the interventions [45-48,52], and the option to personalize the home page was available in one [52]. None of them included any type of social networking feature.

A total of 8 interventions were designed to be accessed in a sequential manor, in which modules were to be completed in a predetermined order. One intervention [46] allowed participant’s free navigation of the system, meaning participants could access and complete modules in any order they decided. However, the recommendation was to work through the modules in the suggested order. One intervention did not report format [36].

### Adherence to Protocol, Usage Data and Satisfaction

Adherence was calculated as a percentage of those randomized to the intervention that also completed postassessment. Nine RCTs reported adherence data, adherence ranged between 48% and 100% (M=82.6%, SD=17.8) and adherence to control ranged between 53.1% and 100% (M=83.4%, SD=16.4%) the control group selected was the active treatment not WLC.

A total of 7 RCTs reported data usage. The data reported varied. Pots et al [52] reported the mean number of completed modules. Carlbring et al [51] reported the number of participants who did not complete any modules, 16% in the CBT condition, and 6% in the ACT condition. Jones et al [36] reported website usage; usage in the ACT condition was significantly higher compared with the control condition (21.7 minutes per login vs 9.4 minutes). Lappalainen et al [46] reported average time spent per week, 50% of participants spent less than one hour a week, 38.9% spent 1-2 hours and 11.1% spent more than 2 hours. Levin et al [47] reported the percentage of participants completing each module, 85% completed lesson 1 and 55% completed lesson 2. Trompetter et al [50] reported module completion, 72% completed 6 modules and 66.2% completed all 9 modules. Levin et al [48] reported a summary of program usage across both intervention and control conditions, 92% completed both modules and spent on average 81.98 minutes using the program.

Satisfaction with treatment is also of importance for adherence and engagement within a Web context. Six RCTs reported a measure of participant satisfaction, of which, 2 focused on system usability [36,45-48,50].

### Meta-Analysis

The number of studies used in the between-group meta­­­-analyses were k=10, k=7, and k=8 for the depression, anxiety, and quality of life outcome measures, respectively. For the within-group meta-analyses, these were k=9, k=7, and k=8.

Studies generally presented the mean and standard deviation of participants’ scores in each outcome measure. The one exception to this was Jones et al [36] whose data on the depression outcome measure was dichotomized, meaning it was not possible to calculate Hedges’ *g* directly. Thus Jones et al [36] was included in the between-group summary effect size calculation but could not be included in the within-group category, as the odds ratio is undefined for this category.

With regard to the between-group summary effect sizes, the effect size for the depression outcome was small and in favor of ACT with *g*=0.24. This was also shown to be statistically significant with *P*=.02 as was the proportion of heterogeneity attributed to true effects, *I*^2^= 55%. The effect size for anxiety was statistically significant. However, it fell short of the lower limit for small effect size with *g*=0.18, *P*=.03. Heterogeneity for this outcome measure did not reach statistical significance *P*=.49, with the same being true for both the effect size and heterogeneity for the quality of life outcome measure. Summary effect sizes belonging to the between-group category are shown in Multimedia Appendix 6.

**Figure 3:**
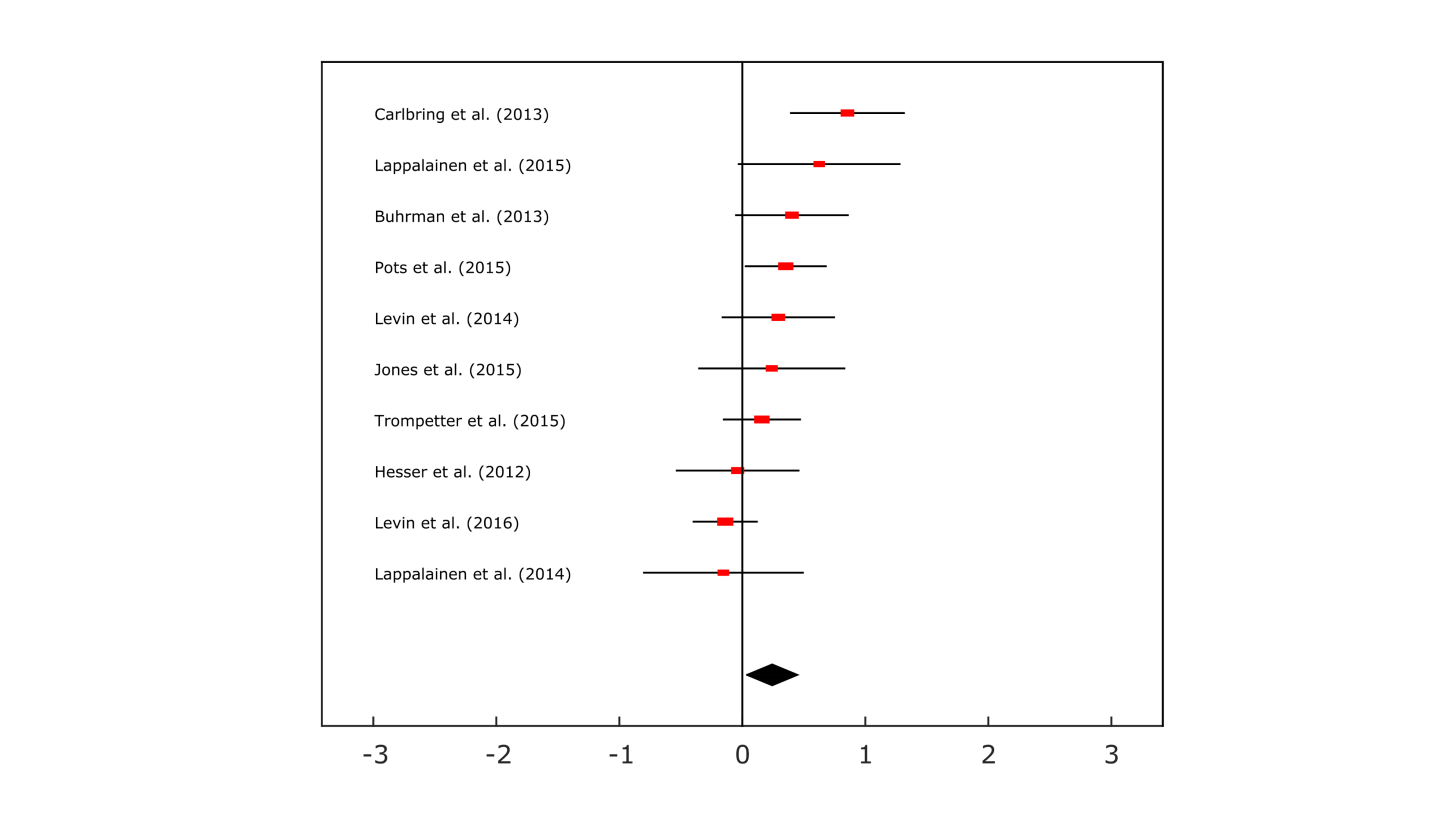
Forest plot produced in MATLAB for the depression outcome measure in the between-group meta-analysis. The x-axis units are in Hedges’ g.

However, the within-group category demonstrated that participants’ scores improved greatly between time points over all outcomes, as displayed in Multimedia Appendix 7. Effect sizes for both the depression and anxiety outcomes were medium in magnitude with *g*=0.73 and 0.51, and the quality of life outcome attained a small effect size of *g*=0.44, all of which were statistically significant *P*=.001; *P<*.001 respectively. The depression and anxiety outcomes also indicated high proportions of heterogeneity between studies with *I*^2^ ≥ 75% whereas ,the quality of life outcome showed a medium proportion of heterogeneity, all of which were also statistically significant with *P*<.001. Figure 3 shows the forest plot for the depression summary effect size for ACT interventions versus comparison groups.

It was not possible to calculate any summary effect sizes at follow-up due to a lack of published data.

### Risk of Bias

**Figure 4:**
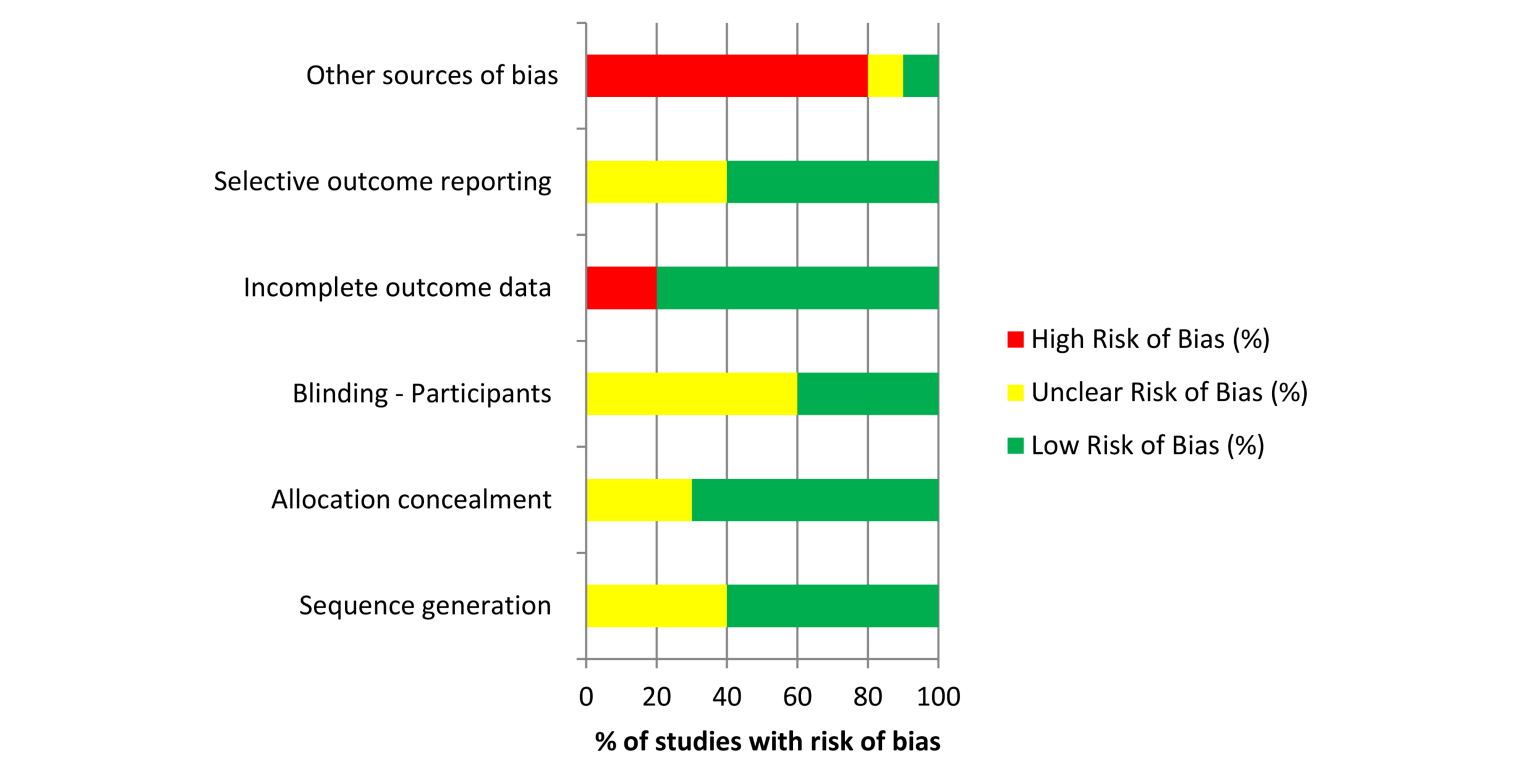
Risk of bias summary per domain.

The risk of bias judgment for each study was made on the information provided in the publication only. Of the 10 studies included, 4 (40%) were judged to be of low risk of bias [29,46,49,52], one (10%) of high risk [36], and an unclear risk of bias was assigned to 5 (50%) [45,47,48,50,51] of the included studies. Figure 4 shows the risk of bias summary.

## Discussion

### Overall Findings

The aim of this study was to examine the published, peer-reviewed literature pertaining to the effectiveness of ACT in the management of CMD and well-being in a Web-based delivery format. Ten RCTs met inclusion criteria. A total of 5 studies focused on depression, 1 on psychological distress, 1 on well-being, 2 on chronic pain, 1 one on tinnitus. All included pre- and post-outcome measures for anxiety, depression, or quality of life. The majority compared against an active comparator or active comparator plus wait list control.

### Principal Results

ACT delivered via a Web-based format is effective for the management of depression (small effect size) and anxiety (which neared the threshold for a small effect size). Web-based ACT interventions were not found to be effective for improving quality of life. Due to lack of published data effect size was not calculated for follow-up effects.

Findings support ACT in a Web-based delivery format for the management of depression. Review of those RCTs focused on depression as the primary outcome [36,45,46,49,51] revealed that ACT was more effective than control, where control was either WLC or an active control. For example, Carlbring et al [51] reported a large effect size and 25% of participants experienced clinical recovery posttreatment. Low dropout and good engagement were reported. On average, participants completed 5 of the 7 modules. Lappalainen et al [45,46] reported Web-delivered ACT was effective over and above face-to-face delivery and WLC with differential outcomes identified in favor of the Web-delivered intervention. Furthermore, treatment effects were maintained at follow-up although they did level out in-line with the WLC. Pots et al [52] reported significant effect of ACT compared to both WLC and an expressive writing condition that were maintained at follow-up. Finally Jones et al [36], while not finding a statistically significant difference, reported positive benefit of ACT over attention control conditions coupled with good user satisfaction for the intervention in a depressed, smoking population. This nonstatistical finding was likely limited by the use of only one depression screening question. All of these studies identified a need for further research and each was limited by small sample sizes. Current findings are in line with those of prior reviews of ACT, Öst [22] reported that ACT was “possibly efficacious for depression.” Five studies primarily targeting depression were reviewed of which, none were Web-based, and three were compared with treatment as usual. Comparing the current effect size for depression against Öst’s [22] overall effect size for active comparators (across all subsamples) the current findings are in-line (*g*=0.24) however this compares less favorably for overall WLC comparison (*g*=0.63) but are in-line with those reported by Ruiz (*g*=0.27) [20].

The effect size for anxiety was statistically significant, although it only neared the threshold for a small effect size (*g*=0.18). There are a number of reasons that might contribute an explanation to this observation of lower effect size. None of the included interventions were primarily designed to treat anxiety; in each case the secondary outcome measure was included. In addition, where pretreatment anxiety scores were low, this limited potential improvement. Furthermore, only 7 of the RCTs included an outcome measure that could be included in the effect size calculation for anxiety. However, Pots et al [52] reported that ACT had positive outcomes for anxiety as well as depression. In prior review findings when ACT was compared with CBT, effect sizes for anxiety did not meet statistical significance [20]. Sharp [23] reviewed ACT specifically for use with anxiety disorders and concluded that data provided preliminary support.

ACT was not found to be effective in delivering improvements in quality of life. However, as with anxiety, none of the interventions specifically targeted quality of life and 2 did not include an outcome measure that could be included in the meta-analysis calculation [36,47]. Both of the RCTs reported by Levin et al [47,48] were focused on feasibility of ACT in an undergraduate population and contained the fewest number of ACT components of all interventions reviewed (the intervention was detailed to be underdevelopment and acknowledged it contained fewer elements). The most recent one of these reported equivalence in outcomes for ACT to the attention control website and lower program usage. However, their analysis of usage patterns suggested that those who engaged more with ACT experienced more positive outcomes and increased psychological flexibility. This was in comparison with their earlier findings that suggested strong acceptability and feasibility. The limited focus on quality of life or well-being is in line with findings identified by Öst [22], where no included studies used them as the primary outcome measure. However in the review by Ruiz [20] an effect size (*g*=0.25) was reported which is higher than that found in the current data. This could be attributable to a few things arguably, while quality of life and well-being can be compared, they are not necessarily the best fit.

Variation in intervention features, delivery format (guided or automated), and study context warrants discussion. For example, The Levin et al studies [47,48] included fewer modules that were available for a shorter period of time, did not conduct preassessment screening and participants received a financial reward for their participation. Jones et al [36] unguided intervention included one preassessment depression screening question. Opportunities for improvement, detection of disorders and effect sizes may be underestimated, due to use of screening questionnaires that have not been validated at baseline. Lappalainen et al [45,46] in contrast utilized multiple therapists to guide participants. Guided interventions, using CBT, are associated with higher effect sizes.

The primary goal of ACT is to improve psychological flexibility. Psychological flexibility in the context of ACT has been defined as “the measure of how a person (1) adapts to fluctuating situational demands, (2) reconfigures mental resources, (3) shifts perspective, and (4) balances competing desires, needs, and life domains” [54]. Examination of ACT specific outcome measures identified improvements in psychological flexibility. Jones et al [36] reported significant improvements in willingness to experience physical triggers and a trend toward willingness to experience emotional triggers (AIS); Lappalainen et al [45,46] reported significant effect in mindfulness skills and psychological flexibility in both face-to-face and Web-based delivery of ACT (using AAQ-II) as did Pots et al [52] with the exception of improvements on the mindfulness facet. However Levin et al [47,48] did not find any significant effect on FFMQ measure or AAQ-II but did report improvements in ACT knowledge. Trompetter et al [50] reported significant improvements at three- and six-month follow-up (FFMQ-SF) and 6 months on PIPS. Although mixed, these findings suggest support for ACT in improving psychological flexibility.

It is important to consider Web-based delivery format in its own right, this is in light of the recent expansion of interest into this field (ACT), evidenced by the multitude of protocol and feasibility studies identified during this review and through the ACBS website.

The secondary aim of this review was to report rates of adherence to Web-based interventions employing ACT as the therapeutic approach. Poor adherence to Web-based mental health interventions is of widespread concern and is well documented in the literature [53,55-57]. Poor adherence has the potential to limit effectiveness [58] and reduce cost effectiveness [59] which is a key benefit of this delivery format. The mean rate of adherence to protocol (82.6%) was higher than published means for CBT based interventions where dropout rates range from 2% to 83% [60] and was comparable to the rates of adherence for the control groups (83.4%). Adherence was calculated as a percentage of those randomized to the intervention and completed postassessment. However reported rates of completion rates remained lower (68.4%). Four interventions [47,48,50,52] specified that they included stakeholders in the design and development process to address adherence, or specifically employed persuasive design features in a bid to encourage adherence and engagement. For example, Pots et al [52] incorporated persuasive technology (but did not specify which) in the design of their intervention, adherence and engagement was reported to be high (73% of participants completed all 9 modules); whereas Levin et al [47] utilized a “tunneled” format. Tunneling refers to using the computerized system to guide users through the therapeutic content in a predefined order, ensuring opportunity to persuade along the way [61]. Such initiatives may help to promote and increase adherence. Couper et al [62] report that higher engagement with the intervention program and Web-based materials is associated with increased likelihood of adherence to study protocols, follow-up data collection points and importantly, changes in health behavior (fruit and vegetable intake study); Strecther [63] reported similar findings in a smoking cessation study where quit rates increased for each additional webpage opened. Although these studies did not employ ACT, it is feasible that the same association could be important across all therapeutic interventions delivered via a Web-based format. Indeed, Trompetter et al [50] noted the increasing need to address this issue in a Web-based context.

### Limitations

There are several limitations to note, such as the variety of outcome measures used in each RCTs may limit the usefulness of the findings. For example, on the quality of life effect size measure 2 studies did not report any outcome measures and a further 2 used a Symptom Checklist 90 (SCL-90) which is considered a measure of well-being as opposed to quality of life. Furthermore, a higher score on the SCL-90 represents lower well-being as opposed to Quality of Life Inventory (QOLI) and Mental Health Continuum Short Form (MHC-SF) where a higher score represents higher quality of life. However, this difference in scoring was adjusted for in the statistical analysis.

Due to lack of published data for follow-up measures, a follow-up effect size could not be calculated.

Due to the small number of included RCTs, meta-analysis by design (eg, WLC or active comparator) was not possible. Of the 10 RCTs reviewed, 3 included WLC, the remainder used either an attention control website, alternative intervention (CBT, moderated discussion forum and expressive writing), or face-to-face delivery format. In the instance where more than one comparator group data was available [29,50,52] we compared against the active treatment. This decision was taken for a number of reasons. Firstly, the majority of included RCTs used active comparators so this was in line with the other comparisons drawn. Secondly, active comparators represent the most realistic real world alternative. For example, CBT as a Web-based therapeutic treatment has a strong and well-established evidence base [15,64] and interventions using this approach are freely available (eg, *MoodGYM*) for the treatment of depression and other CMD as are psycho-education websites and online discussion groups. We acknowledge that the diversity of comparators may limit the generalizability of our findings. However, we feel this is a practical and useful approach to adopt in the current review.

One study [51] was based on behavioral activation and ACT and as such, the effects of the treatment intervention cannot exclusively be attributed to ACT.

The interpretation of effect size magnitude (both between- and within-group) used in this paper adheres to Cohen’s rule-of-thumb. However, concerning the interpretation of within-group effect sizes, some authors prefer to opt for an alternative classification [65]. Here small effects are classified as 0.5 ≤ *g*<0.8, medium effects as 0.8 ≤ *g*<1.1 and large effects as *g* ≥ 1.1. When this classification is applied to the 3 within-group meta-analyses conducted in this paper then the effect sizes for the depression and anxiety outcomes of *g*=0.73 and 0.51 are recategorized as small, while the effect size for the quality of life outcome of *g*=0.44 does not reach the threshold for small effect size. However, statistical significance remains unaffected in all the 3 cases.

Usage data were not reported in all RCTs, in the future standardized reporting is recommended including agreement on completion rate. For example, Karyotaki et al [66] advocate intervention completion to be defined as, completion of 75% or more of the total modules. Finally, publication bias and the trend to report positive results over negative or neutral results must be taken into consideration when reviewing the results of the current meta-analysis. It is possible that our results are overestimated as a result. We reviewed all relevant ACT RCTs listed on the ACBS website to identify any further studies not cited in our database searches.

### Implications for Practice

Web-based delivery of interventions has undergone a recent expansion in a range of health contexts, physical health, mental health, and lifestyle behavior change. This brings with it new considerations for the effective delivery of therapeutic interventions. Increased interest in this delivery format stems from the explosion in access to affordable personal mobile devices that offer easy access to the Internet from all locations with 3G or Wi-Fi coverage. Such easy and convenient accessibility is thus one of the key advantages of this new delivery format, coupled with the cost effectiveness associated with its ability to facilitate widespread reach access across the population.

Further research into the use of ACT via Web-delivery, is required to continue to explore its effectiveness and to understand the most effective components for this delivery context. Specifically those targeting anxiety and well-being would be of benefit as positive well-being continues to grow as an area of public interest and means to promote and prevent poor mental health. Studies should focus on recruiting larger populations to avoid concerns with lack of statistical power and to ensure wider generalizability of findings. Studies should also seek to examine the long-term effect of ACT through inclusion of follow-up periods in future RCTs. In this systematic search, 9 protocol documents and 5 feasibility studies were identified suggesting that this is a developing field of interest and that evidence will expand in coming years. Thus, we provide a first review of the evidence.

Risk of bias assessment concluded that the majority of studies had a low or unclear risk of bias and thus there is potential for future studies to ensure that they continue to report in line with Cochrane recommendations to ensure the availability of best quality evidence.

It is worth noting that the intervention used in Carlbring et al [51] has since been utilized in subsequent RCT [67] but where it is described predominately as iCBT. Thus, this study would not have been identified in the current search strategy and, if it had been identified through other sources, would have been excluded at title-abstract stage due to variations in reporting of the intervention components and its stated theoretical perspective. While a sensitivity analysis, where we included this study did not alter our findings (Multimedia Appendix 8), this did highlight a wider concern in this research field. Future research into Web-based interventions, using all types of therapy should consider the way in which they report and describe the intervention and treatment. Consistent reporting of the intervention across RCTs would allow assessment of any differences in effectiveness of these types of programs (CBT, ACT), which are clearly distinguished in the field of psychology in terms of their mechanisms of action. This would also facilitate the systematic review process as research in this area develops.

In addition, analyses of ACT-specific outcome measures are a potential for further exploration.

### Conclusion

ACT is efficacious for the treatment of depression in a Web-based delivery format. Further research is required across all mental health and emotional well-being domains to continue to develop and review the evidence base in this delivery format. This review adds strength to this evidence base, across delivery formats.

## Appendix

Multimedia Appendix 1 Keyword search terms.

Multimedia Appendix 2 Main results table.

Multimedia Appendix 3 Trial arms and comparators.

Multimedia Appendix 4 Outcome measures utilized in each RCT.

Multimedia Appendix 5 The various key statistics relating to the summary between-group effect sizes by outcome measure. From left to right these are: k number of studies; g summary effect size with confidence interval (CI); Z test statistic; p-value relating to g; Q-statistic for total dispersion between studies; I^2-statistic for proportion of dispersion due to true effects; and p-value relating to I^2.

Multimedia Appendix 6 The key statistics relating to the summary within-group effect sizes by outcome measure.

Multimedia Appendix 7 Table of interventions by core processes.

Multimedia Appendix 8 Meta-analysis results additional permutations.

## Abbreviations

ACBS: Association for Contextual Behavioral Science
ACT: Acceptance and commitment therapy
CMD: common mental disorder
CBT: cognitive behavior therapy
PTSD: Post-Traumatic Stress Disorder
OCD: obsessive compulsive disorder
WLC: wait list control
RCT: Randomized controlled trial
HADS: Hospital and depression scales
BDI: Beck Depression Inventory
BAI: Beck Anxiety Inventory
DASS: Depression Anxiety Stress Scales
CES-D: Center for Epidemiologic Studies Depression Scale
QOLI: Quality of Life Inventory
GHQ-12: General health Questionnaire 12 items
SCL-90: Symptom checklist 90 items
MHC-SF: Mental health Continuum Short Form
AIS: Avoidance and Inflexibility Scale
AAQ-II: Acceptance and Action Scale
FFMQ: Five Facet Mindfulness Questionnaire
PIPS: Psychological Inflexibility Scale
ELS: Engaged Living Scale
