# Supplementary figures and images for: Effectiveness of Web-Delivered Acceptance and Commitment Therapy in Relation to Mental Health and Well-Being: A Systematic Review and Meta-Analysis
Source: J Med Internet Res. 2016 Aug 24;18(8):e221. doi: 10.2196/jmir.6200 (PMC5039035; doi:10.2196/jmir.6200)

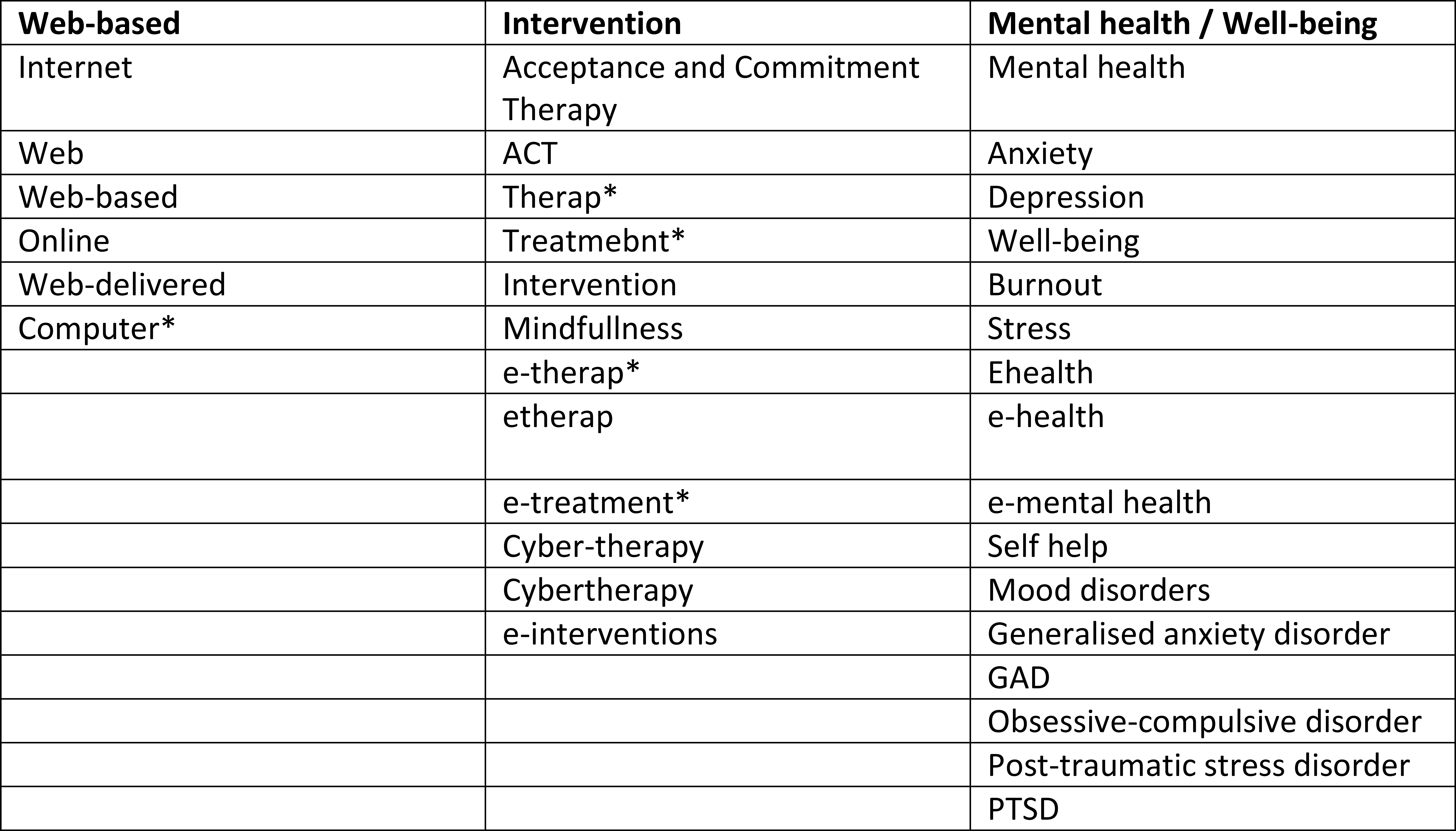

Supplement: Multimedia Appendix 1 [file jmir_v18i8e221_app1.png]

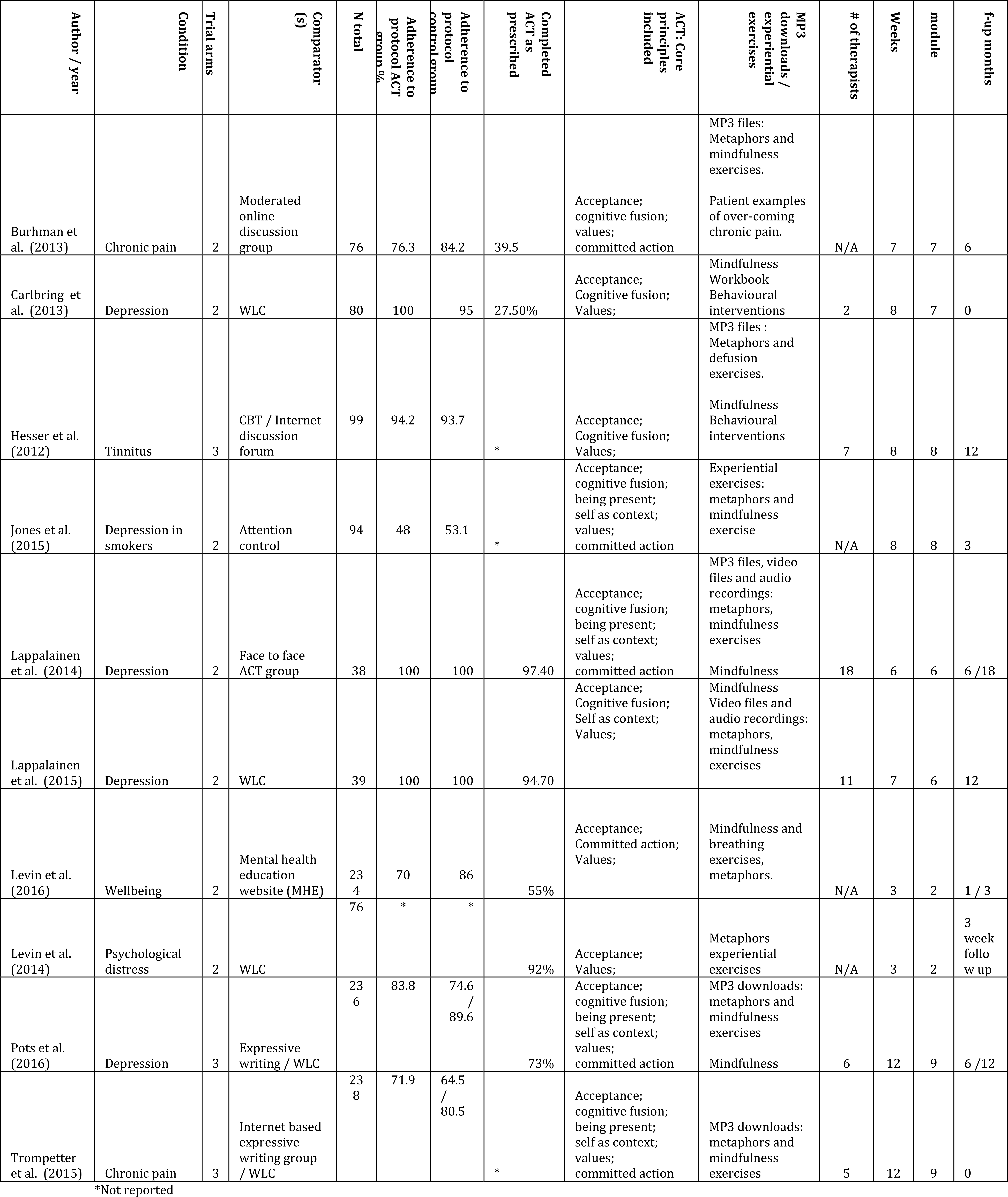

Supplement: Multimedia Appendix 2 [file jmir_v18i8e221_app2.png]

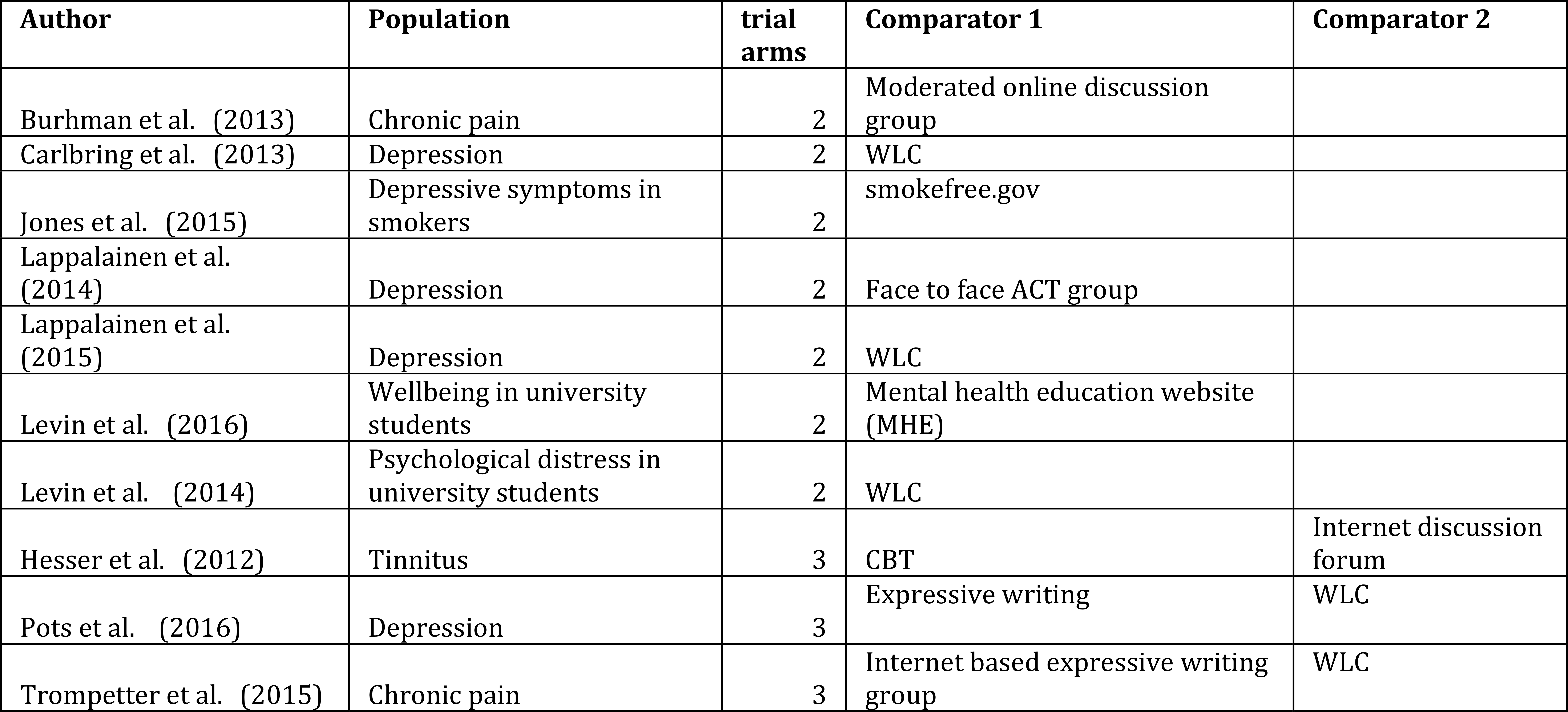

Supplement: Multimedia Appendix 3 [file jmir_v18i8e221_app3.png]

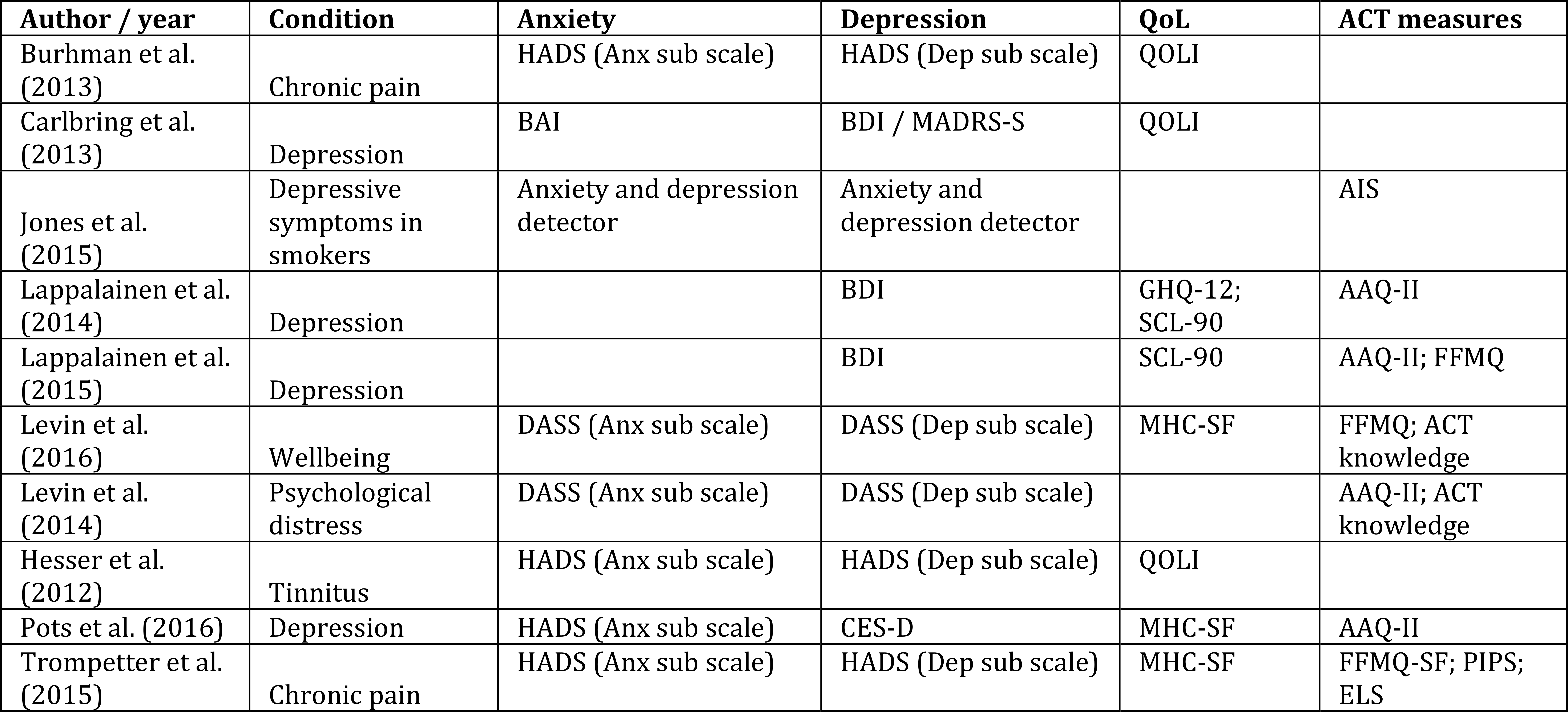

Supplement: Multimedia Appendix 4 [file jmir_v18i8e221_app4.png]

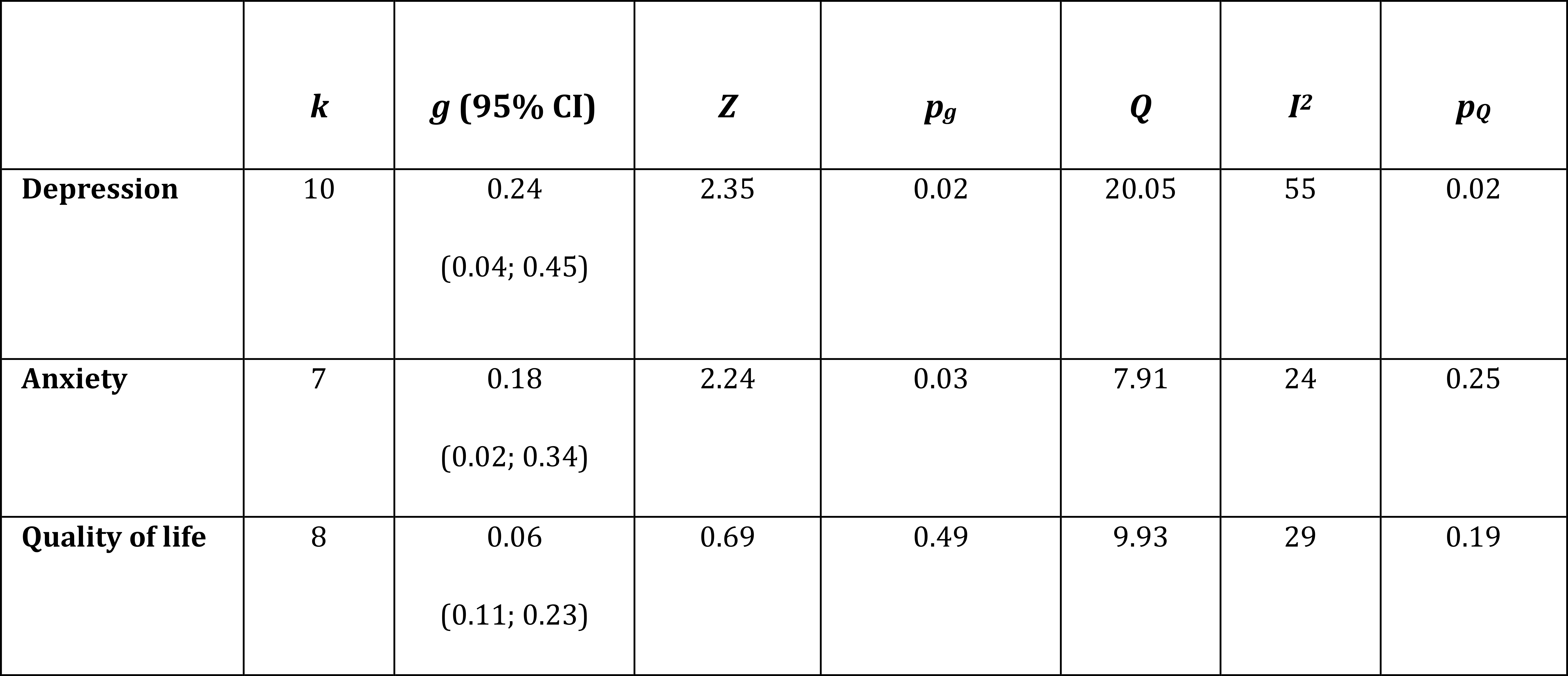

Supplement: Multimedia Appendix 5 [file jmir_v18i8e221_app5.png]

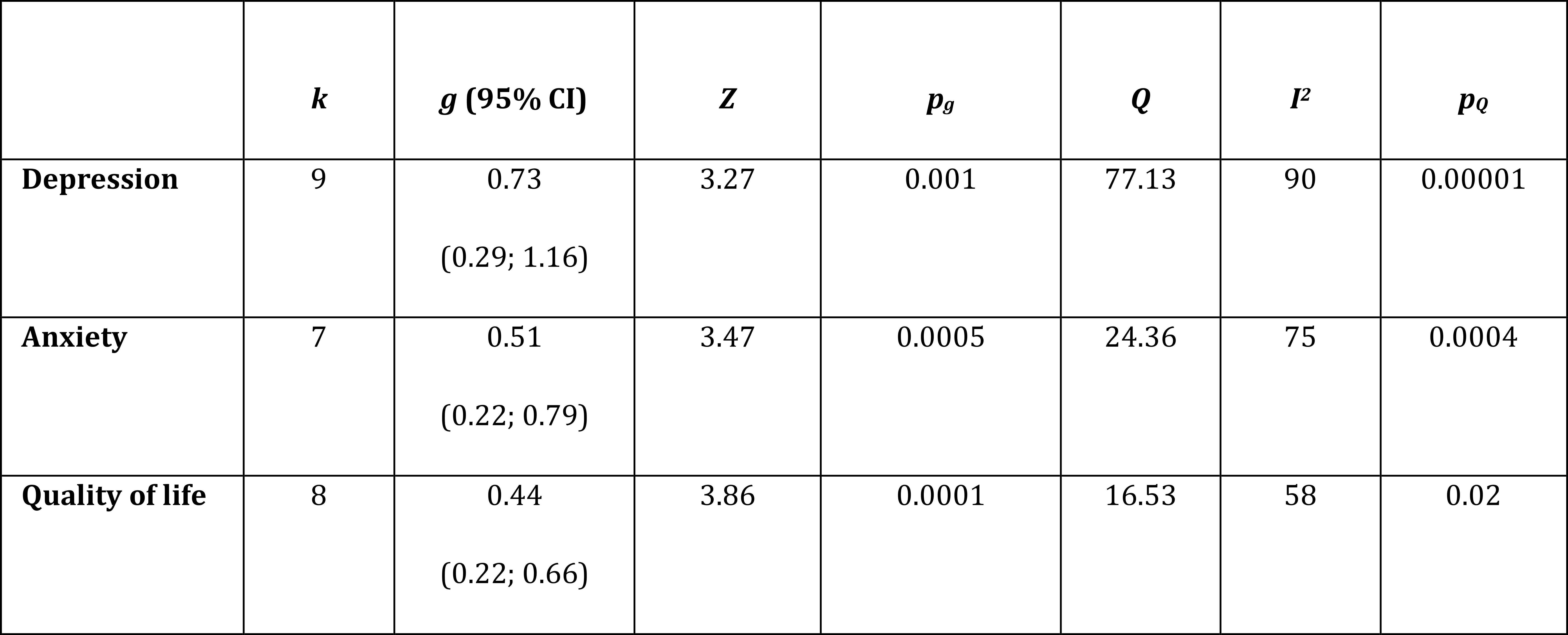

Supplement: Multimedia Appendix 6 [file jmir_v18i8e221_app6.png]

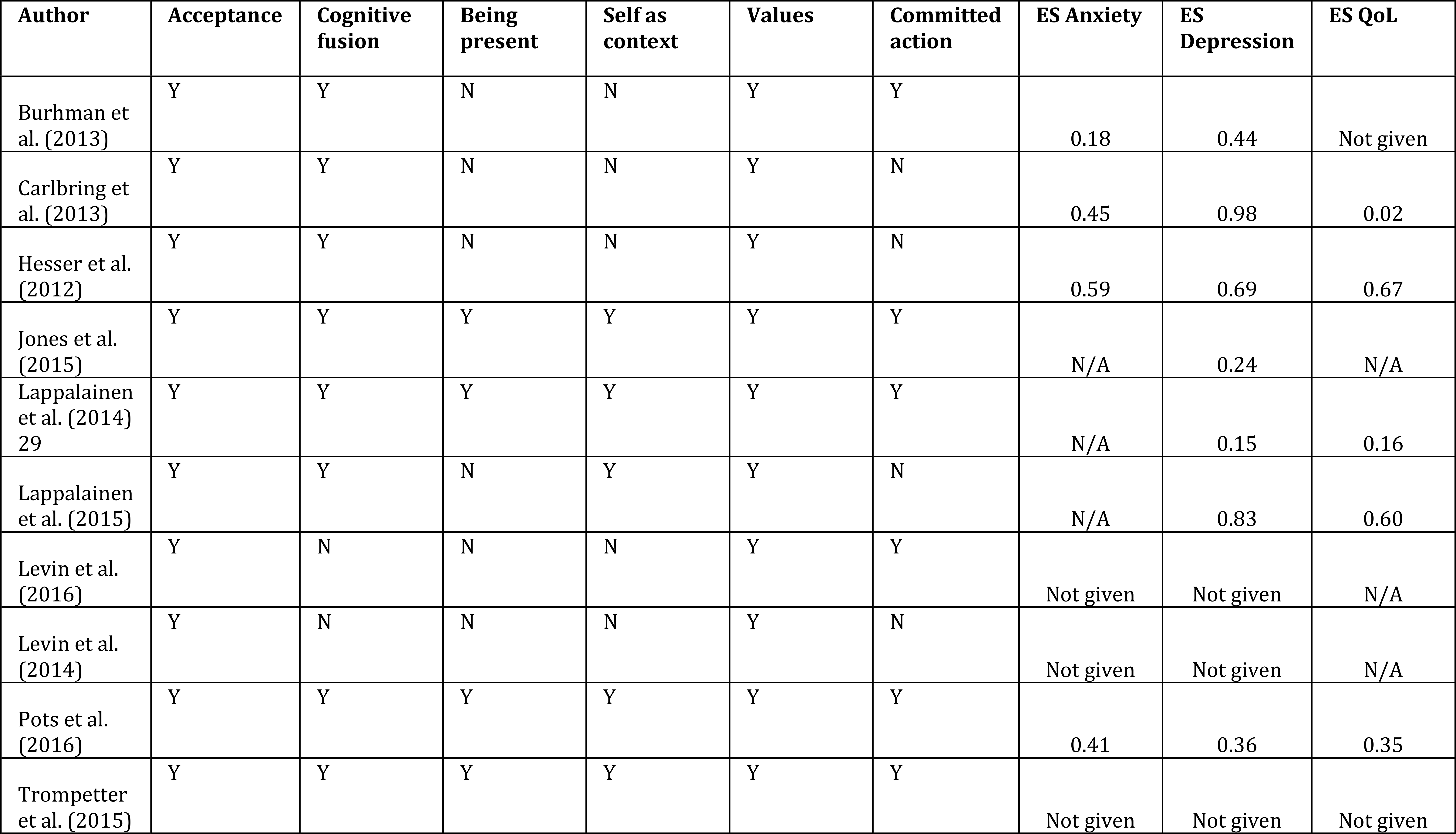

Supplement: Multimedia Appendix 7 [file jmir_v18i8e221_app7.png]

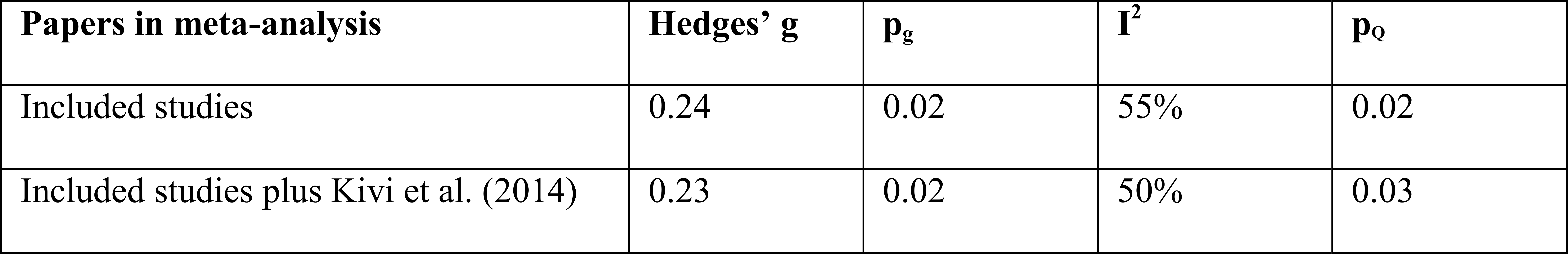

Supplement: Multimedia Appendix 8 [file jmir_v18i8e221_app8.png]
